# Supplementary figures and images for: Cellular Delivery of Functional AntimiR Conjugated to Bio-Produced Gold Nanoparticles
Source: Noncoding RNA. 2025 Sep 11;11(5):66. doi: 10.3390/ncrna11050066 (PMC12452297; doi:10.3390/ncrna11050066)

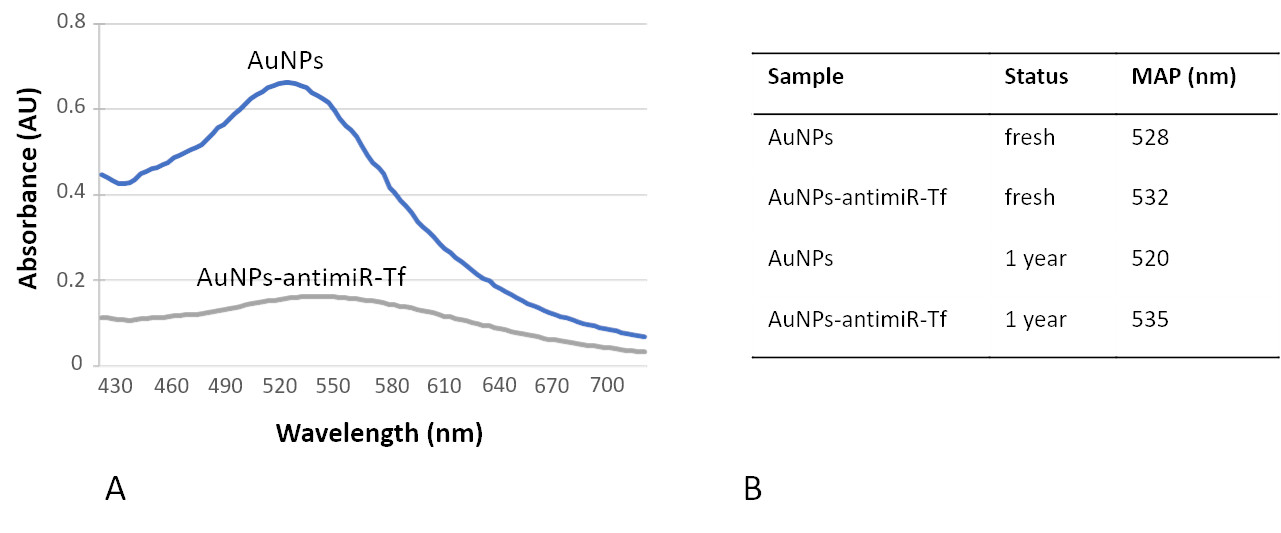

Supplement: Supplementary file 1 [file ncrna-11-00066-s001.zip › ncrna-3747494-supplementary.jpg]
